# Genetic variant panel allows predicting both obesity risk, and efficacy of procedures and diet in weight loss

**DOI:** 10.3389/fnut.2023.1274662

**Published:** 2023-11-16

**Authors:** Alejandra Mera-Charria, Francisco Nieto-Lopez, Manel Pacareu Francès, Priscila Marques Arbex, Laura Vila-Vecilla, Valentina Russo, Carolina Costa Vicente Silva, Gustavo Torres De Souza

**Affiliations:** ^1^Dorsia Clinics, Madrid, Spain; ^2^Catedra UCAM Dorsia, Catholic University San Antonio of Murcia, Guadalupe, Spain; ^3^Independent Researcher, Barcelona, Spain; ^4^Fagron Genomics US, LLC, Austin, TX, United States; ^5^Fagron Genomics, Barcelona, Spain; ^6^Fagron (Netherlands), Rotterdam, Netherlands

**Keywords:** obesity, weight loss, genetics, single nucleotide polymorphism, bariatric surgery

## Abstract

**Purpose:**

Obesity is a multifactorial condition with a relevant genetic correlation. Recent advances in genomic research have identified several single nucleotide polymorphisms (SNPs) in genes such as FTO, MCM6, HLA, and MC4R, associated with obesity. This study aimed to evaluate the association of 102 SNPs with BMI and weight loss treatment response in a multi-ethnic population.

**Methods:**

The study analyzed 9,372 patients for the correlation between SNPs and BMI (dataset A). The correlation between SNP and weight loss was accessed in 474 patients undergoing different treatments (dataset B). Patients in dataset B were further divided into 3 categories based on the type of intervention: dietary therapy, intragastric balloon procedures, or surgeries. SNP association analysis and multiple models of inheritance were performed.

**Results:**

In dataset A, ten SNPs, including rs9939609 (FTO), rs4988235 (MCM6), and rs2395182 (HLA), were significantly associated with increased BMI. Additionally, other four SNPs, rs7903146 (TCF7L2), (rs6511720), rs5400 (SLC2A2), and rs7498665 (SH2B1), showed sex-specific correlation. For dataset B, SNPs rs2016520 (PPAR-Delta) and rs2419621 (ACSL5) demonstrated significant correlation with weight loss for all treatment types. In patients who adhered to dietary therapy, SNPs rs6544713 (ABCG8) and rs762551 (CYP1A2) were strongly correlated with weight loss. Patients undergoing surgical or endoscopic procedures exhibited differential correlations with several SNPs, including rs1801725 (CASR) and rs12970134 (MC4R), and weight loss.

**Conclusion:**

This study provides valuable insights into the genetic factors influencing BMI and weight loss response to different treatments. The findings highlight the potential for personalized weight management approaches based on individual genetic profiles.

## 1 Introduction

Obesity is a complex condition resulting from an imbalance between energy intake and expenditure. Affecting over 2 billion people worldwide, it has significant negative impacts on quality of life and health, including an increased risk of numerous comorbidities such as type 2 diabetes, cardiovascular disease, and cancer. It is important to also consider the intricate interplay between the increase in adipose tissue and the modifications in it that contribute to the development of insulin resistance and type 2 diabetes (1, 2). Obesity treatment necessarily involves lifestyle changes; however, surgical and endoscopic procedures may also be necessary. Understanding the multifactorial causes of obesity, including genetic and environmental factors, is crucial for effective treatment and prevention strategies (3). The genomic markers related to the development of obesity are essential in understanding and predicting this condition. Nevertheless, there is also evidence showing that there are loci related to weight loss in response to the treatment of choice (3–6).

Nutrigenomics is an interdisciplinary field that examines the intricate interactions between genetic and dietary factors on health outcomes. By combining genetics, nutrition, and molecular biology, researchers aim to understand how individual genetic variations influence a person’s response to nutrients, food components, and dietary patterns. The goal of nutrigenomics research is to provide personalised dietary recommendations and promote optimal health outcomes. This emerging field has significant potential for improving health and preventing chronic diseases such as type 2 diabetes, obesity, and cardiovascular diseases by tailoring dietary interventions to the specific patient genotype (7–11).

This approach has garnered considerable attention in clinical research due to its potential for personalising nutritional recommendations based on an individual’s genetic profile. For instance, individuals with variations in the FTO gene have been found to be more susceptible to obesity and may benefit from personalised dietary interventions (8, 12, 13). Nutrigenetic testing has also been employed to identify individuals at risk of developing lactose intolerance, coeliac disease, and phenylketonuria, providing them with tailored dietary management. These examples illustrate the potential of nutrigenetics to improve clinical outcomes by enabling more targeted and effective nutritional interventions (7, 11).

A genome-wide association study (GWAS) conducted on over 21,000 subjects from the Taiwan Biobank identified several significant adiposity-associated trait-loci pairs. Among these genes, RALGAPA1 was found to be a specific genetic predisposing factor for high BMI in the Taiwanese population. Additionally, the study detected a moderate genetic correlation between waist-hip ratio (WHR) and BMI, indicating that different genetic determinants exist for abdominal adiposity and overall adiposity. Intriguingly, the study also uncovered the importance of neural pathways that might influence body fat percentage (BF%), waist circumference (WC), and WHR in the Taiwanese population. These findings suggest that multiple genetic factors contribute to obesity and BMI in the Taiwanese population, necessitating further research to better understand the specific pathways involved (14).

In a study by Locke et al., the researchers investigated the heterogeneity of BMI-associated SNPs’ effects, observing stronger effects in women for two SNPs near SEC16B and ZFP64 and significant heterogeneity between European and African-descent samples for two SNPs near NEGR1 and PRKD1, as well as between European and East Asian individuals for an SNP near GBE1. The majority of BMI-associated SNPs demonstrated comparable effects across ancestries and sexes. Utilizing LD differences across populations, the study fine-mapped association signals, revealing that the inclusion of non-European individuals in the meta-analysis refined the genomic region or reduced the credible set’s SNPs for 10 of the 27 BMI loci fine-mapped on Metabochip (5, 6, 15).

Examining the combined effects of lead SNPs at 97 loci in an independent European-descent sample (*n* = 8,164), the study found an average increase of 0.1 BMI units per BMI-increasing allele and a significant mean BMI difference between individuals with the most and least BMI-increasing alleles. Additionally, incorporating genetic risk scores into a model with age, sex, and four genotype-based principal components marginally yet significantly enhanced obesity prediction accuracy (5, 6, 15).

Subsequent evaluations analyzed the association between BMI and approximately 2.8 million SNPs in up to 123,865 individuals, confirming 14 known obesity susceptibility loci and identifying 18 new loci, including a copy number variant near GPRC5B. Notably, several loci were situated near hypothalamic energy balance regulators (e.g., MC4R, POMC, SH2B1, BDNF) and an incretin receptor (GIPR), potentially providing novel insights into human body weight regulation (16). Overall, these findings suggest that obesity is a complex trait influenced by a combination of genetic and environmental factors. Nevertheless, there is clear genetic contribution to the development of obesity and alterations in body composition. Therefore, finding and employing genomic markers to predict and better understand this pathology might be of great help in dealing with patients presenting this condition.

Genetic variants seem to play a crucial role in the outcomes of bariatric surgeries, such as Roux-en-Y gastric bypass (RYGB). A diverse range of weight loss responses has been observed among patients, and several genes have been associated with these differences. The 15q26.1 locus near ST8SIA2 and SLCO3A1 has been significantly linked to weight loss after RYGB, with the expression of ST8SIA2 in omental fat at baseline correlating with post-surgery weight loss. Additionally, genes such as PKHD1, HTR1A, NMBR, and IGF1R have been identified as potential contributors to the variation in weight loss outcomes after RYGB. However, genetic risk scores (GRSs) for adiposity and the rs1358980-T risk allele in the VEGFA locus did not predict weight loss after gastric bypass surgery in one study. The association between a GRS for abdominal obesity and the response to bariatric surgery may be dependent on the association between the GRS and baseline BMI. Overall, these findings highlight the importance of considering genetic factors when evaluating weight loss outcomes following bariatric surgeries, as they may significantly influence individual responses (17–19).

While there have been previous studies that investigate the impact of genetic factors on the efficacy of surgical and endoscopic interventions for obesity, these studies often involve limited sample sizes or narrow scopes. Consequently, there remains a significant need for comprehensive evaluations that encompass larger, multi-ethnic populations. The identification of specific genetic variations associated with obesity and the predisposition to respond favourably to various therapeutic approaches serves a dual purpose. Firstly, it aids in elucidating the underlying biological mechanisms that contribute to weight loss following therapeutic interventions. Secondly, it provides valuable insights that could facilitate personalized medical decision-making, enabling clinicians to predict patient response to different treatments with greater accuracy. In this context, our study aims to fill the existing gaps in the literature by providing a broader evaluation of the role of genetic variations in influencing the effectiveness of weight loss therapies.

Additionally, it is significant to emphasise that there are numerous factors which ought to be taken into consideration when deciding upon the clinical approach to a patient, whether through a reversible procedure, such as an intragastric balloon, or a non-temporary one, like bariatric surgery or endoscopic interventions. While guidelines exist for the selection of patients for each type of procedure, several of the clinical markers overlap, thus posing a considerable challenge in evaluating the risk versus benefit ratio (20, 21). In the current study, the efficacy of each type of procedure was correlated with the genotypes present in several genomic markers with the aim to enhance the decision-making process for the choice between temporary and non-temporary procedures (20–22).

Therefore, comprehending the genetic factors involved in obesity, high BMI, and the weight loss process is crucial for a more in-depth understanding of these processes and determining optimal management strategies. In this study, we analysed two patient databases, which were genotyped for a panel of 102 SNPs. These SNPs were identified as significant in various energy metabolism pathways, as well as genes implicated in the behavioural aspects of eating.

## 2 Methods

### 2.1 Study design

We analysed two distinct databases containing matched genotypes of patients for the 102 SNPs examined, in relation to either their BMI at a single time point or weight loss and BMI changes in patients who underwent weight loss treatment. The complete data comprised two sets of patient information: (A) 9,372 patients whose genotypes were matched to their BMI at the time of DNA collection; (B) 474 patients who were both genotyped and had their weight and BMI assessed before and after follow-up for dietary treatment, endoscopic intragastric balloon procedures, or surgical interventions.

The datasets are composed of patients who had been previously genotyped as part of their nutritional counselling, *as per* request of the patient for the treatment offered to them, and had their BMI and anthropometric data recorded on their medical record in a fully anonymised database. Only the genotype and BMI data were recovered with no identifiable features associated. Both datasets were fully anonymised before any data processing, ensuring that only the BMI, weight loss, and genotype for each patient were compiled, according to the dataset. These patients had their genotypes and clinical data stored as medical records within a repository, which included individuals from a diverse range of ethnic backgrounds.

### 2.2 Genotyping procedure

Briefly, all the genotyping assays were performed by qPCR from genomic DNA previously collected from the patients. The DNA was extracted with the automated bead-based method using the KingFisher system (Thermo Fisher). Nucleic acid were quantified with the RNAseP method in order to ensure sample quality control. The SNPs analysed are summarized on Table 1. All SNP genotyping was done with the Taqman probe assay designed on the Genotyping software offered by the Thermo Fisher platform, the raw qPCR amplification data was updated to the cloud service to ensure genotyping to be performed automatically.

**Table 1:** Summary of genomic information of the SNPs analysed in patients included in datasets A and B.

SNP	Chromosome	Gene or region involved	SNP	Chromosome	Gene or region involved	SNP	Chromosome	Gene or region involved
rs671	12	ALDH2	rs7498665	16	SH2B1	rs2383208	9	CDKN2A, CDKN2B
rs1050450	3	GPX1	rs7903146	10	TCF7L2	rs7756992	6	CDKAL1
rs1800566	16	NQO1	rs9939609	16	FTO	rs7901695	10	TCF7L2
rs4680	22	COMT	rs1121980	16	FTO	rs12970134	18	MC4R
rs4880	6	SOD2	rs1800588	15	LIPC	rs17782313	18	MC4R
rs1051168	15	NMB	rs7799039	7	LEP	rs4788102	16	SH2B1
rs1726866	7	TAS2R38	rs1800546	9	ALDOB	rs1395479	4	AGA
rs1800497	11	DRD2	rs76917243	9	ALDOB	rs891684	17	SLC39A11
rs2229616	18	MC4R	rs17700633	18	MC4R	rs2025804	1	LEPR
rs6277	11	DRD2	rs10811661	9	CDKN2A/B	rs5400	3	SLC2A2
rs1801282	3	PPAR-Y	rs676210	2	APOB	rs2241201	12	MMAB
rs662799	11	APOA5	rs12740374	1	CELSR2	rs3846663	5	HMGCR
rs2016520	6	PPARD	rs2650000	12	HNF1A	rs12934922	16	BCMO1
rs713598	7	TAS2R38	rs6511720	19	LDLR	rs7501331	16	BCMO1
rs2470893	15	CYP1A1	rs6544713	2	ABCG8	rs1279683	20	SLC23A2
rs762551	15	CYP1A2	rs2395182	6	HLA	rs33972313	5	SLC23A1
rs10766197	11	CYP2R1	rs4713586	6	HLA	rs10741657	11	CYP2R1
rs2282679	4	GC	rs7454108	6	HLA	rs12785878	11	NADSYN1, DHCR7
rs1550532	2	DGKD	rs7775228	6	HLA	rs2060793	11	CYP2R1
rs1570669	20	CYP24A1	rs182549	2	MCM6	rs3829251	11	NADSYN1, DHCR7
rs17251221	3	CASR	rs4988235	2	MCM6	rs12272004	11	INTERGENIC
rs1801725	3	CASR	rs1800562	6	HFE	rs964184	11	ZNF259, LOC100128347, APOA5, SIK3, BUD13
rs7481584	11	CARS	rs3811647	3	TF	rs1042714	5	ADRB2
rs780094	2	GCKR	rs4820268	22	TMPRSS6	rs1801133	1	MTHFR
rs11577390	1	AMY1-AMY2	rs10850219	12	KCTD10	rs4343	17	ACE
rs4244372	1	AMY1	rs11144134	9	TRPM6	rs4654748	1	NBPF3
rs1799883	4	FABP2	rs13146355	4	SHROOM3	rs602662	19	FUT2
rs17300539	3	ADIPOQ	rs3925584	11	DCDC5	rs8177253	3	TF
rs1805134	1	LEPR	rs4072037	1	MUC1	rs9770242	7	NAMPT
rs2289487	15	PLIN1	rs1056836	2	CYP1B1	rs1501299	3	ADIPOQ
rs2419621	10	ACSL5	rs1048943	15	CYP1A1	rs2241766	3	ADIPOQ
rs5883	16	CETP	rs1695	11	GSTP1	rs1800629	6	TNF-α
rs490683	3	GHSR	rs174547	11	FADS1	rs1800795	7	IL-6
rs5082	1	APOA2	rs2237892	11	KCNQ1	rs1800896	1	IL-10

### 2.3 Association analyses and statistical framework

The association between genotype and clinical features was analysed according to the data group. In dataset A, patients’ genotypes were associated with their BMI at the time of DNA collection. The dataset B was composed of patients who were treated with one of the weight loss therapeutic interventions studied. The patients were categorised into groups based on clinical, surgical, or endoscopic interventions for weight loss. Subsequently, the genotypes were associated with weight and BMI loss. Additional data were also evaluated for significance; in dataset B, the follow-up duration until the last weight measurement was assessed, patients were not enrolled to the studied, as it was a retrospective medical record analysis, therefore, there was no information related to the beginning of the intervention. The baseline BMI information was employed to infer weight loss when comparing to the final results. For dataset A, SNP association was tentatively performed for subsets of male and female patients; however, since no relevant data were found, this will not be discussed further.

Statistical analyses were performed using R (version 4.2.2) as the computational environment. The SNPassoc package was chosen for the necessary calculations (23). We evaluated the genotype–phenotype association without restrictions to the dominance model and applied Bonferroni correction. The dominance models applied were: (1) codominance, in which alleles exhert effect; (2) dominance, SNP allele is dominant in determining the phenotype; (3) recessive, SNP allele is recessive the determination of the phenotype; (4) overdominant, heterozygous does not determine a phenotype within the two homozygous possibilities; and (5) additive, neither allele is dominant. The *p*-values reported are the ones depicting the highest statistical correlation. We aimed to assess the clinical and molecular significance according to the specific study of each SNP. Statistical significance was defined as *p* < 0.01.

## 3 Results

### 3.1 Association between SNPs and BMI

**Figure 1:**
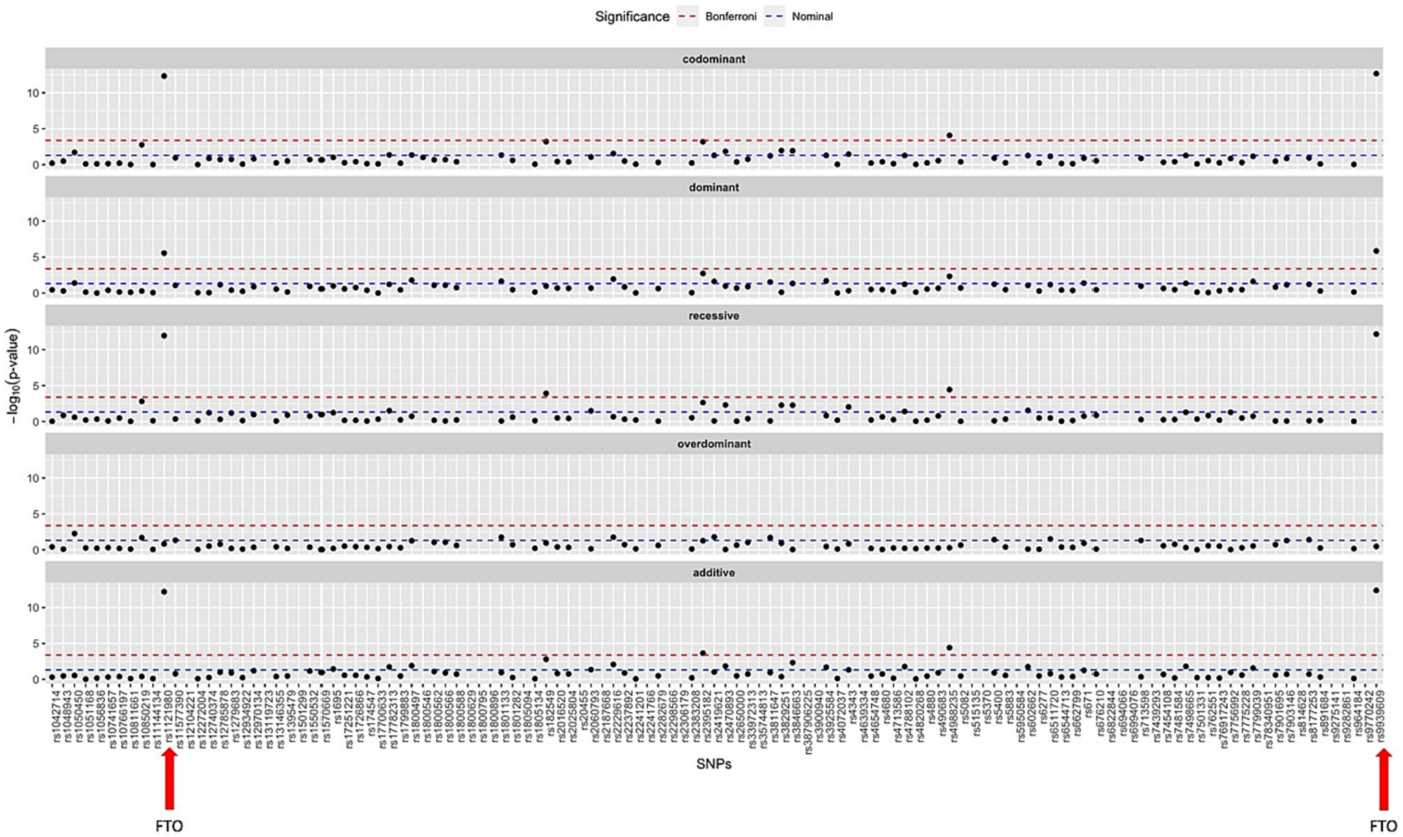
Manhattan plot summarizing the statistic results for all SNPs studied under all models of association to the BMI as phenotype. The SNPs studied in the FTO gene, found to be the most significant ones, are marked with a red arrow.

An SNP association analysis was conducted to correlate BMI with genotype for the studied SNPs. Upon analysing the 9,372 patients belonging to dataset A, ten SNPs demonstrated statistically significant correlations with increased BMI. Since the raw genotype data were generated by qPCR for a limited number of SNPs, the genotyping rate for all SNPs was 100%, as only patients with complete genotypes were considered for this study. Table 2 summarises the *p*-values found for each SNP under the evaluated model of genotype association and Figure 1 displays the Manhattan Plot for visualization of the results.

**Table 2:** *p* values obtained upon statistic evaluation of the association between BMI (phenotype) to the 102 SNPs analysed.

SNP	Genic region	*p* value for codominant model	*p* value for dominant model	*p* value for recessive model	*p* value for overdominant model	Log-additive
rs9939609	FTO	2.114E-13	1.3648E-06	6.9133E-13	0.3409127	6.1171E-13
rs1121980	FTO	4.6083E-13	2.6843E-06	1.1519E-12	0.1531425	9.2509E-13
rs4988235	MCM6	8.2181E-05	0.00466017	3.782E-05	0.5219651	3.9791E-05
rs182549	MCM6	0.0006154	0.10589411	0.0001213	0.11425821	0.00161272
rs2395182	HLA	0.00063581	0.00187173	0.00239678	0.05458373	0.00023214
rs10850219	KCTD10	0.00167209	0.5343385	0.00162645	0.0202502	0.4217869
rs3829251	NADSYN1	0.01063817	0.77429347	0.00541801	0.12041572	0.44187628
rs3846663	HMGCR	0.01149159	0.04712569	0.00548761	0.92405252	0.0047282
rs2470893	CYP1A1	0.01441176	0.10656919	0.00510184	0.8942208	0.01444574
rs1050450	GPX1	0.0193709	0.03977497	0.26727718	0.00532972	0.30002542
rs2229616	MC4R	0.0280324	0.011384	0.22106289	0.01769627	0.00857146

### 3.2 Correlation between SNPs and female patients

Interestingly, four additional SNPs were found to have statistically significant associations only among the female population of dataset A. Among female patients, SNPs in the LDL receptor (LDLR), transcription factor TCF7L2, glucose transporter protein SLC2A2, and SH2B1 were positively associated. The following *p*-values were found for the SNPs under all different models of inheritance: rs7903146 (TCF7L2) *p* = 0.0027, rs6511720 (LDLR) *p* = 0.003, rs5400 (SLC2A2) *p* = 0.003, and rs7498665 (SH2B1) *p* = 0.008.

### 3.3 Follow-up length of patients under weight loss therapy

In dataset B, which comprised patients who underwent either dietary therapy or surgical or endoscopic procedures, these patients were monitored to assess weight loss. The overall mean follow-up time for the patients in dataset B was 188.72 days. Among patients exclusively receiving dietary intervention, the mean time was 123.97 days. However, for patients monitored for their diet, 58 were assigned zero days of follow-up in the dataset, indicating that there was no adherence to the treatment at all. Consequently, the rate of adherence to the follow-up program for patients previously tested with this panel of SNPs was 78.27%.

### 3.4 SNPs on the PPAR-delta and ACSL5 genes predict weight loss in all patients accompanied

**Figure 2:**
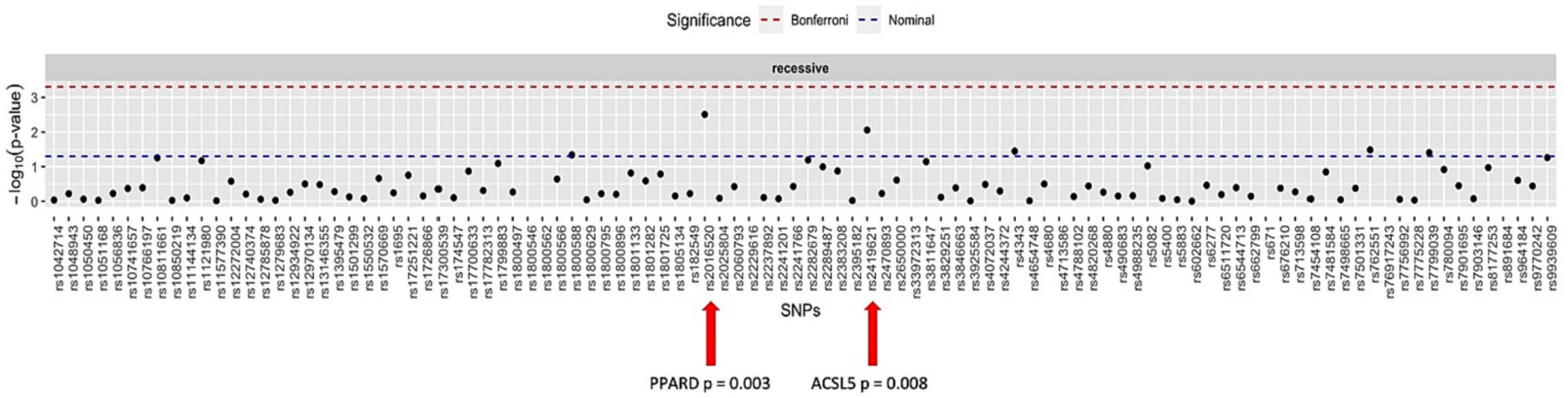
Manhattan plot showing the distribution of *p*-values for the association between the genotypes and weight loss in all patients.

When considering all patients in dataset B, who were treated for weight loss, including those who underwent dietary intervention, intragastric balloon procedures, or surgeries, the SNPs rs2016520 and rs2419621 demonstrated significant correlation between genotype and weight loss using the recessive model. Figure 2 summarises the data found for this dataset.

### 3.5 Response to diet therapy

Weight loss was documented in patients in dataset B who followed appropriate dietary management and were monitored by nutrition professionals. The difference in BMI and weight was strongly correlated with the genotypes of two SNPs: rs6544713 (*p* = 0.00001) and rs762551 (*p* = 0.00033). It is also worth noting that the SNP studied in the COMT enzyme, rs4680, exhibited some degree of correlation when using the recessive model of association, with a *p*-value of 0.02.

### 3.6 SNP-association to the weight loss in patients subject to surgical or endoscopic procedures

Weight loss in patients undergoing surgical or endoscopic procedures was greater, as expected and supported by the literature (24, 25). Moreover, several SNPs could also be differentially correlated with these procedures.

Patients who underwent intragastric balloon procedures demonstrated a positive correlation between weight loss and the genotype of the following SNPs: rs1801725 (CASR gene) *p* = 0.00131, rs4820268 (TMPRSS6) *p* = 0.002, rs17300539 (ADIPOQ) *p* = 0.003, and rs17251221 (CASR) *p* = 0.007.

The difference in weight before and after endoscopic sleeve or POSE procedures exhibited a high degree of statistical significance with 5 SNPs. The genotypes of the following SNPs were correlated using a recessive model to the amount of weight lost: rs12970134 (MC4R) *p* = 0.00003, rs17782313 (MC4R) *p* = 0.00003, rs4072037 (MUC1) *p* = 0.00052, rs1550532 (ACE) *p* = 0.001, and rs1550532 (DGKD) *p* = 0.00078. It is also worth mentioning that rs4343, in the leptin gene, showed a *p*-value of 0.02. Furthermore, the PPAR-delta SNP rs2016520 has been associated with weight loss in patients who underwent all types of procedures, including balloon, endoscopic sleeve, i.e., and POSE.

## 4 Discussion

In this study, we analysed two groups of patients, separate into: dataset A (9,372 patients), and dataset B (474 patients). We aimed to investigate the association between selected SNPs and BMI, as well as their potential role in predicting weight loss response to various treatments in a diverse population. Our findings revealed that several of the studied SNPs were significantly correlated with BMI. We also identified a subset of SNPs that were uniquely associated with BMI in female patients. Moreover, we observed that specific SNPs were significantly linked to weight loss outcomes in patients undergoing different weight loss treatments. These results highlight the complex interplay between genetic factors and obesity, as well as the response to weight loss interventions.

Furthermore, the difference in potential genomic markers that predict increased BMI and weight loss in different procedures highlights the importance of nutrigenetics in the management of patients to control obesity and its comorbidities (7). By understanding the genetic predispositions and individual responses to weight loss interventions, we can develop personalized treatment approaches that optimize weight loss outcomes and overall health. Table 3 summarises the findings.

**Table 3:** Summary of the *p*-values found for the association between genotypes and the phenotypes.

BMI (all patients)			BMI (female patients)			Weight loss (all patients)			Weight loss (diet group)			Weight loss (balloon group)			Weight loss (sleeve or POSE group)			Weight loss (sleeve, POSE or balloon group)		
SNPid	Gene Regio	*p*-value	SNPid	Gene Regio	*p*-value	SNPid	Gene Regio	*p*-value	SNPid	Gene Regio	*p*-value	SNPid	Gene Regio	*p*-value	SNPid	Gene Regio	*p*-value	SNPid	Gene Regio	*p*-value
rs9939609	FTO	6.9133E-13	rs7903146	TCF7L2	0.0027	rs2016520	PPARD	0.00309	rs6544713	ABCG8	0.00874	rs1801725	CASR	0.00131	rs12970134	MC4R	0.00003	rs2016520	PPARD	0.00840
rs1121980	FTO	4.6083E-13	rs6511720	LDLR	0.003	rs2419621	ACSL5	0.00874	rs762551	CYP1A2	0.00001	rs4820268	TMPRSS6	0.002	rs17782313	MC4R	0.00003			
rs4988235	MCM6	3.782E-05	rs5400	SLC2A2	0.003				rs4680	COMT	0.00033	rs17300539	ADIPOQ	0.003	rs4072037	MUC1	0.00052			
rs182549	MCM6	0.0001213	rs7498665	SH2B1	0.008							rs17251221	CASR	0.007	rs1550532	ACE	0.001			
rs2395182	HLA DQ2.2	0.00187173													rs1550532	DGKD	0.00078			
rs10850219	KCTD10	0.00162645													rs4343	LEP	0.02			
rs3829251	NADSYN1	0.00541801																		
rs3846663	HMGCR	0.00548761																		
rs2470893	CYP1A1	0.00510184																		
rs1050450	GPX1	0.00532972																		
rs2229616	MC4R	0.011384																		

Moreover, the strong interaction between the two SNPs on the FTO gene, rs9939609 and rs1121980, and increased BMI as a phenotype reinforces the importance of the FTO gene in the development of obesity, as previously reported in the literature (26–28). FTO has been associated with leptin concentration, as well as alterations in HDL and triglyceride levels (29, 30). These SNPs have also been linked to dietary intake, leptin levels, and body mass distribution (31, 32). However, our study did not find any association between these SNPs and weight loss in any of the intervention groups. This finding suggests that while the FTO gene may play a significant role in the development of obesity, its involvement in weight loss response might be limited or influenced by other factors. Our results warrant further investigation to better understand the underlying mechanisms.

The process of losing weight involves creating a caloric deficit to restore the energy balance. However, biochemical and psychological factors can determine the success of sustaining the caloric deficit to achieve significant weight loss. This has led to the search for genomic markers to predict the ideal type of diet based on individual factors. Personalising the diet through nutrigenomics could be a successful approach to improving weight loss outcomes (4).

The relevance of genetics in predicting diets and weight loss has become increasingly apparent, as recent advances in genomics have enabled researchers to uncover key genes that influence individual responses to specific dietary interventions. Understanding these genetic factors can help tailor personalised nutrition plans, ultimately leading to more effective weight loss and better overall health outcomes. Among the most significant genes related to diet and weight loss are FTO, MC4R, and PPARγ, which have been implicated in the regulation of appetite (4, 33), energy expenditure, and fat metabolism. Studies employing Mendelian randomisation have further strengthened the causal links between these genes, dietary patterns, and obesity risk. As our understanding of the complex interplay between genetics and diet continues to grow, nutrigenomic approaches will likely play an increasingly important role in developing effective, personalised weight loss strategies (4, 34).

Apart from the dietary and life-style approach, surgical and endoscopic methods do also play an important role in aiding patients achieve significative weight loss. Nevertheless, the efficacy of those methods is also variable, which has to be balanced with the risks involved in the procedures (24, 25). Therefore, the search for genomic markers of success related to surgical, endoscopic, and dietary interventions seem an interesting possibility to guide the decisions.

In this study, SNPs rs1050450 (GPX1) and rs2229616 (MC4R) have been associated with increased BMI and body weight. Both genes have previously been linked to obesity (35), and it is noteworthy that MC4R is involved in appetite regulation (36–38). Although the GPX1 gene does not show any apparent functional connection to weight gain, our results support previous findings. The presence of variations in the melanocortin receptor could serve as a crucial marker for determining patient management, as it indicates both the risk of obesity and a predisposition to poor appetite control. Consequently, appropriate nutritional intervention may be guided by the genotype.

In the female population, the SNP rs5400 (SLC2A2) was associated with increased BMI. In addition to corroborating previous studies (39), this finding offers valuable insights for designing nutrigenomic diets for both weight loss and type 2 diabetes management. This SNP indicates that individuals may have altered sensitivity to sugar, leading to higher sugar intake (40, 41). The rs7498665 in the SH2B1 gene has also been found to be associated with BMI in our female subset (31, 42–44).

Apart from variations previously associated with BMI, we also found a positive correlation between BMI and SNPs previously linked to alterations in lipid and lipoprotein levels: rs10850219 (KCTD10) (45, 46); rs3846663 (HMGCR) (33, 47, 48); and rs6511720 (LDLR) (49–51).

The SNP rs2470893, which influences the function of the CYP1A1 enzyme, has also been found to be associated with an increase in BMI. Although there appears to be no direct mechanistic link, a previous study has shown this polymorphism to be associated with both alterations in coffee consumption and BMI. Researchers speculated that this connection might be due to differences in appetite control, including variations in circulating levels of asprosin (52).

Another intriguing correlation of interest was discovered in the MCM6 gene. The two SNPs studied in this gene, rs4988235 and rs182549, were strongly linked with alterations in body mass, in our study. Variants in the lactase gene are widely known to cause lactose intolerance, which, *per se*, has no direct correlation to weight gain (53). A few studies have shown the rs4988235 SNP to be linked to changes in body composition and lipid concentrations (35, 54). The rs182549 has also been correlated with changes in food consumption patterns (55) These previous findings, combined with the strong connection to BMI increase observed in our data, suggest that this is a relevant target for nutrigenetic management. Variations in the MCM6 also drive changes in the microbiota, which can potentially be therapeutically manipulated (56).

With regards to the genotype–phenotype correlations discovered in dataset B of this study, we identified several genomic markers capable of determining predispositions for success under specific therapeutic approaches to weight loss. Variations in the ABCG8 and COMT genes (SNPid rs6544713), previously linked to cholesterol levels (57), and in the COMT gene, widely recognised for its relevance in the behavioural aspects of eating (58, 59), have been associated with weight loss in patients exclusively undergoing dietary therapy. By understanding the implications of alterations in eating patterns, dietitians may be able to further tailor diets for patients presenting alterations in the COMT gene, who are predisposed to behavioural changes.

Among the SNPs associated with success in intragastric balloon procedures, the genotypes of rs17300539 in the adiponectin gene were positively correlated. Variations in the adiponectin gene have been previously linked to obesity, likely due to altered expression of this marker (60, 61). In our study, we identified the SNP rs17300539 as a novel genomic marker correlated with weight loss. In patients undergoing sleeve or POSE surgery, SNPs in the melanocortin receptor were strongly associated with the capacity to lose weight. This finding offers both an interesting target for nutrigenetic management and a mechanistic understanding of weight gain and loss in light of the central regulation of appetite. In addition, the rs4343 in the leptin gene, previously associated with weight gain and satiety (62, 63), was a novel finding related to weight loss in patients undergoing these procedures.

The discovery of markers linked to the success of treating patients via either a temporary procedure, i.e., the intragastric balloon, or a definitive surgical or endoscopic intervention, contributes significantly to the existing body of literature. It offers the potential to more effectively guide physicians in determining the optimal therapeutic approach. From a biological perspective, it is noteworthy that polymorphisms in the adiponectin gene were correlated with success in patients treated with the intragastric balloon. This association could potentially be attributed to the role of adiponectin in mediating signals from adipose tissue, which is involved in insulin resistance, glucose level regulation, and ultimately, energy metabolism (64–66). Previous studies have linked other adiponectin gene polymorphisms to the response to aerobic exercise (67).

Simultaneously, polymorphisms in the melanocortin-4 receptor and leptin genes were linked to successful outcomes in non-temporary procedures, namely surgical or endoscopic interventions. This suggests an inherent correlation between the response to these procedures and the central mechanisms of appetite control. In light of these novel findings, the utilisation of genomic markers could supplement the clinical data used in the decision-making process to determine the most appropriate type of procedure (3, 68).

In addition to the findings related to genetic analysis, we also observed interesting results regarding the overall utility of this approach. In dataset B, 78.27% of patients demonstrated a global adherence to dietarian changes, which is considerably higher compared to studies with non-genotyped patients (1, 2, 69, 70). This suggests that nutrigenetic tools might also impact the efficacy of diet due to increased adherence.

## 5 Conclusion

This study sheds light on the complex interplay between genetic factors and obesity, as well as the response to weight loss interventions. The identification of significant correlations between specific SNPs and BMI, along with SNPs uniquely associated with BMI in female patients, underscores the importance of considering personalized treatment approaches based on individual genetic profiles. The findings also reveal the potential of nutrigenetics in guiding patient management by targeting key genes, such as FTO, GPX1, MC4R, SLC2A2, and SH2B1. These results contribute to our understanding of the genetic predispositions and individual responses to weight loss interventions, enabling the development of tailored treatment strategies to optimize weight loss outcomes and overall health.

Moreover, the study highlights the potential impact of nutrigenetic tools on the efficacy of diet due to increased adherence. The higher global adherence to diet observed among genotyped patients suggests that incorporating genetic information into weight loss interventions may lead to improved patient engagement and success. As obesity and its comorbidities continue to pose significant public health challenges, the insights gained from this research pave the way for more targeted and effective approaches to weight management, ultimately leading to better health outcomes for patients.

## Data availability statement

The original contributions presented in the study are included in the article, further inquiries can be directed to the corresponding author.

## Ethics statement

Ethical approval was not required for the studies involving humans because the whole study was conducted solely with data obtained from a database of medical records and genetic data from patients previously collected. All the data is anonymised completely so as to comply with not needing ethical approval. The studies were conducted in accordance with the local legislation and institutional requirements. The human samples used in this study were acquired from a by-product of routine care or industry. Written informed consent to participate in this study was not required from the participants or the participants’ legal guardians/next of kin in accordance with the national legislation and the institutional requirements.

## Author contributions

AM-C: Writing – original draft, Writing – review & editing. FN-L: Conceptualization, Data curation, Writing – original draft. MF: Conceptualization, Writing – review & editing. PA: Conceptualization, Formal analysis, Supervision, Writing – original draft. LV-V: Conceptualization, Investigation, Writing – original draft, Writing – review & editing. VR: Formal analysis, Investigation, Methodology, Writing – original draft. CS: Investigation, Validation, Writing – review & editing. GS: Data curation, Formal analysis, Investigation, Methodology, Project administration, Writing – original draft, Writing – review & editing.
